# Construction and validation of a decision tree for treating metabolic acidosis in calves with neonatal diarrhea

**DOI:** 10.1186/1746-6148-8-238

**Published:** 2012-12-06

**Authors:** Florian M Trefz, Annette Lorch, Melanie Feist, Carola Sauter-Louis, Ingrid Lorenz

**Affiliations:** 1 Clinic for Ruminants with Ambulatory and Herd Health Services at the Centre for Clinical Veterinary Medicine, LMU Munich, Sonnenstrasse 16, 85764, Oberschleissheim, Germany; 2 UCD School of Veterinary Medicine, University College Dublin, Belfield, Dublin 4, Ireland

**Keywords:** D-lactate, Clinical signs, Calves, Neonatal diarrhea, Treatment protocol, Sodium bicarbonate, Intravenous fluid therapy

## Abstract

**Background:**

The aim of the present prospective study was to investigate whether a decision tree based on basic clinical signs could be used to determine the treatment of metabolic acidosis in calves successfully without expensive laboratory equipment. A total of 121 calves with a diagnosis of neonatal diarrhea admitted to a veterinary teaching hospital were included in the study. The dosages of sodium bicarbonate administered followed simple guidelines based on the results of a previous retrospective analysis. Calves that were neither dehydrated nor assumed to be acidemic received an oral electrolyte solution. In cases in which intravenous correction of acidosis and/or dehydration was deemed necessary, the provided amount of sodium bicarbonate ranged from 250 to 750 mmol (depending on alterations in posture) and infusion volumes from 1 to 6.25 liters (depending on the degree of dehydration). Individual body weights of calves were disregarded. During the 24 hour study period the investigator was blinded to all laboratory findings.

**Results:**

After being lifted, many calves were able to stand despite base excess levels below −20 mmol/l. Especially in those calves, metabolic acidosis was undercorrected with the provided amount of 500 mmol sodium bicarbonate, which was intended for calves standing insecurely. In 13 calves metabolic acidosis was not treated successfully as defined by an expected treatment failure or a measured base excess value below −5 mmol/l. By contrast, 24 hours after the initiation of therapy, a metabolic alkalosis was present in 55 calves (base excess levels above +5 mmol/l). However, the clinical status was not affected significantly by the metabolic alkalosis.

**Conclusions:**

Assuming re-evaluation of the calf after 24 hours, the tested decision tree can be recommended for the use in field practice with minor modifications. Calves that stand insecurely and are not able to correct their position if pushed require higher doses of sodium bicarbonate, if there is clinical evidence of a marked D-lactic acidosis. In those calves, determining the degree of loss of the palpebral reflex was identified as a useful decision criterion to provide an additional amount of 250 mmol sodium bicarbonate. This work demonstrates the clinical relevance of the discovery that D-lactate is responsible for most of the clinical signs expressed in neonatal diarrheic calves suffering from metabolic acidosis.

## Background

Metabolic acidosis is a frequently observed complication of neonatal diarrhea in calves. Intestinal losses of bicarbonate ions, decrease of glomerular filtration of hydrogen ions as a result of a reduction of renal perfusion and accumulation of L-lactate and other unidentified organic anions were considered to be the cause of this condition in the past [1-3]. In the past decade, scientific work has shown that D-lactate is a major component of high anion gap acidosis in neonatal calf diarrhea [4-6]. More importantly, recent research has demonstrated that most clinical signs of metabolic acidosis are attributable to an increase in blood levels of D-lactate [7,8]. Despite a good correlation between D-lactate and base excess values, levels of D-lactate can vary widely, especially in calves with moderate to severe acidosis [6]. Thus estimation of the degree of metabolic acidosis on the basis of clinical signs seems to be a challenge in bovine practice [9]. Several protocols and “depression scores” for diagnosing and treating metabolic acidosis in bovine field practice have been suggested [10-16]. Alterations in posture and behavior are usually used to determine bicarbonate requirements whereas the suckling reflex and the degree of enophthalmos are thought to be useful clinical tools to decide if intravenous fluid therapy is necessary [11-14]. However, prospective studies which evaluated the success and feasibility of these treatment protocols are currently not available to our knowledge. Simple guidelines for the dosage of sodium bicarbonate relying on posture/ability to stand and degree of dehydration as sole criteria were described in a retrospective analysis of the records of 188 calves with neonatal diarrhea by Lorenz and Lorch [9]. The aim of the present prospective study was to investigate whether a decision tree which is based on these recommendations could be used to determine the treatment of metabolic acidosis without the help of expensive laboratory equipment. Since the theoretically determined outcome of the recommended dosages of sodium bicarbonate for the present study population revealed that overdosing is more likely than underdosing [17], the impact of iatrogenic alkalosis on the success of therapy was also evaluated. Another purpose was to evaluate whether the strong impact of blood D-lactate concentrations on clinical signs influence the therapeutic success and therefore the clinical management of metabolic acidosis in neonatal calf diarrhea.

## Methods

### Animals

For the purpose of this prospective study, 150 calves with a diagnosis of neonatal diarrhea admitted for treatment to the Clinic for Ruminants, LMU Munich, between September, 2009, and April, 2010, were examined. The management of all calves within the study was within standard protocols of the clinic for the treatment of calves with neonatal diarrhea. Therefore no animal ethics approval had to be sought. The exclusion criteria for calves were need for surgical intervention (n = 2), marked hypernatremia (> 170 mmol/l, n = 4), failure to receive the entire determined infusion volume (n = 3), and force-feeding prior to admission in one calf with anorexia since birth. A total of 15 calves admitted with diarrhea were euthanized on arrival (n = 8) or throughout the study (n = 7) on grounds of severe concurrent disease (e.g. gangrene of extremities, meningitis, generalized peritonitis). One calf with peritonitis (at necropsy) died. Data sets of three additional calves that were euthanized at a later point in time were excluded retrospectively because of serious findings at necropsy including peritonitis, BVDV infection and cecocolic intussusception. Thus data for 121 calves remained in the study. Due to geographical reasons most (92%) of the calves belonged to the Simmental breed.

### Clinical and laboratory examination

All physical examinations followed a standardized protocol and were carried out at the time of admission and 24 hours later by the same investigator (FMT). Blood samples were collected from the jugular vein for laboratory analysis at given times. Clinical parameters were categorized and scored as shown in Table 1. Examination of the posture/ability to stand included lifting of the animal if it was not able or willing to stand up. Attempts to lift the calves to their feet were carried out by two persons. The hydration status of each calf was assessed by estimating the degree of enophthalmos and the duration of skin tenting on the upper eyelid. The severity of enophthalmos was additionally quantified by measuring the distance (in mm) between the medial canthus and the eyeball since the degree of dehydration can be easily derived by using the following formula [18]:

(1) $$\%\;\text{dehydration}\;=\;1.91\;*\;\left(\text{enophthalmos},\;\text{mm}\right)\;-\;0.49$$

**Table 1:** Improvement of clinical signs during the study period as indicated by the number of calves in the clinical categories on admission (n = 121) and 24 hours after the initiation of therapy (n = 116)

**Clinical Categories (Score)**	On admission^*^	**After 24 h**	**p value**
	**n**	**n**	
**Posture/Ability to stand**			
Standing up by itself **(1)**	7	12	**< 0.001**
Standing up after encouragement **(2)**	53	99	
Standing securely after lifting **(3)**	7	4	
Insecurely, able to correct position if pushed **(4)**	14	1	
Insecurely, not able to correct position if pushed **(5)**	24 (29)	-	
Sternal recumbency **(6)**	7	-	
Lateral recumbency **(7)**	4	-	
**Behavior**			
Adequate reaction to acoustic and optical stimuli, very bright and alert **(1)**	18	48	**< 0.001**
Adequate reaction to acoustic and optical stimuli **(2)**	37	62	
Delayed reaction to acoustic and optical stimuli **(3)**	42	6	
Calf reacts only to painful stimuli (e.g. venipuncture) **(4)**	15 (20)	-	
No reaction to painful stimuli **(5)**	4	-	
**Suckling reflex**			
Strong **(1)**	33	45	**0.003**
Weak **(2)**	42	52	
Absent or chewing movements **(3)**	41 (46)	19	
**Palpebral reflex**			
Eyelids are closed immediately and fully **(1)**	55	98	**< 0.001**
Eyelids are closed immediately but not fully **(2)**	34	17	
Eyelids are closed with delay and not fully **(3)**	20 (24)	1	
Eyelids are not closed at all **(4)**	7 (8)	-	
**Degree of enophthalmos**^**#**^			
None **(1)**	41	99	**< 0.001**
Slightly sunken **(2)**	31 (32)	13	
Moderately sunken **(3)**	26 (29)	4	
Severely sunken **(4)**	18 (19)	-	
**Degree of skin tenting**			
Resolution of skin tenting prompt **(1)**	8	35	**< 0.001**
Slightly delayed **(2)**	23	45	
Moderately delayed **(3)**	38 (40)	32	
Skin tenting does not resolve spontaneously **(4)**	47 (50)	4	

*Including calves that were not treated with the decision tree for reasons of expected treatment failure are indicated by brackets.

# The bulbi were classified as sunken in cases of a visible gap between the eyeball and caruncula lacrimalis. The median measured eyeball recession (mm) in the four categories were 1, 4, 6, and 9, respectively.

If the general condition of the animals allowed (n = 102), a sample of ruminal fluid was taken using a probe with a perforated brass head. Ruminal pH was measured with an electronic pH meter (testo 230-pH meter, Testo GmbH & Co., Lenzkirch, Germany).

Heparinized blood samples were immediately examined for Base excess, HCO_3_^-^, PCO_2_ and concentrations of sodium, potassium, chloride, and ionized calcium using a blood gas analyzer (Rapidlab® 865 blood gas analyzer, Bayer Vital GmbH, Fernwald, Germany). Blood biochemistry analysis included determination of D-lactate, L-lactate and glucose from heparinized blood samples containing sodium fluoride as glycostatic agent and determination of total protein, urea, creatinine and activity of y-glutamyltransferase from serum samples (Automatic Analyzer Hitachi 911, Roche Diagnostics, Indianapolis, USA). Determination of D-lactate was carried out by using D-lactate dehydrogenase as described by Lorenz et al. [19]. Anion gap (AG) was calculated using the following formula:

(2) $$\text{AG}\;=\;\left[{\text{Na}}^{+}\right]\;+\;\left[{\text{K}}^{+}\right]\;-\;\left[{\text{HCO}}_{3}^{-}\right]-\left[{\text{Cl}}^{-}\right]$$

### Treatment

**Figure 1:**
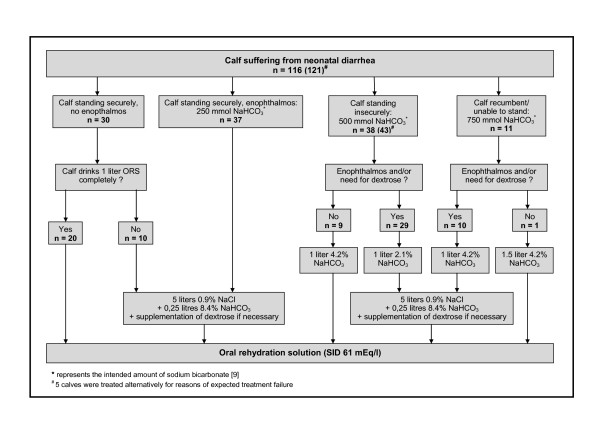
**Assessed decision tree on the basis of recommendations for the dosage of sodium bicarbonate by Lorenz and Lorch**[9]

The amount of sodium bicarbonate administered to the calves was determined with the aid of the decision tree (Figure 1) without regarding individual body weight. The decision tree was created on the basis of simple recommendations on the dosage of sodium bicarbonate relying on posture/ability to stand and degree of dehydration as described in a retrospective analysis by Lorenz and Lorch [9]. Calves that were neither dehydrated nor assumed to be acidemic received an oral rehydration solution (ORS) as sole therapy. In order to prevent any deterioration of the general condition in such calves, the intake of ORS was tested immediately after the initial examination. Calves that did not drink one liter of ORS completely, received a constant drip infusion with 250 mmol sodium bicarbonate. In cases where intravenous correction of acidosis and/or dehydration was deemed necessary, the provided amount of sodium bicarbonate ranged from 250 to 750 mmol (depending on alterations in posture) and infusion volumes from 1 to 6.25 liters. Short-term infusions (by rapidly infusing 1 or 1.5 liter of a 4.2% solution of sodium bicarbonate over a period of 10 to 15 minutes) were carried out in calves showing signs of moderate to severe metabolic acidosis (standing insecurely or unable to stand) but no enophthalmos. In this context, enophthalmos (as a clinical sign of dehydration) was defined as a visible gap between the eyeball and *caruncula lacrimalis*, which corresponded to a measured eyeball recession of at least 3–4 mm. Calves with enophthalmos always received a constant drip infusion consisting of 5 liters of isotonic saline spiked with 250 ml of an 8.4% solution of sodium bicarbonate over the study period of 24 hours. If impairment of ability to stand was evident in such calves, part of the total amount of sodium bicarbonate was rapidly infused beforehand in 1 liter of a 2.1% or 4.2% solution. Commercially available packages (250 ml) of an 8.4% solution of sodium bicarbonate, which were diluted with distilled water to obtain a 2.1% or 4.2% solution if necessary, were used for all treatment procedures.

Dextrose was added to the infusion solutions if one of the following criteria was met:

history of malnutrition or lack of milk intake for more than 24 hours

marked hyperemia of the mucous membranes and severe congestion of the episcleral vessels (as signs of suspected septicemia)

hypothermia on admission (≤ 37°C)

ruminal drinking with concomitant severe ruminal acidosis (pH < 5) as determined above and a high proportion of caseinate in ruminal fluid [20]. In this case, milk and ORS were withheld for two feedings.

In cases where supplementation of dextrose was deemed necessary, a constant drip infusion was performed by replacing 1 liter of isotonic saline by 1 liter of a 20% dextrose solution.

All calves received meloxicam (Metacam®, Boehringer Ingelheim Vetmedica GmbH, Germany) at a dose of 0.5 mg/kg intravenously immediately following admission examination. Since Bavaria is a selenium-deficient area, all calves received a preparation with vitamin E and selenium (0.3 mg/kg sodium selenite and 10 mg/kg vitamin E, SC; Vitamin E-Selen-Lösung®, cp-pharma, Germany).

Considering recommendations for the use of antimicrobials [21,22] as well as potentially useful clinical predictors of septicemia in diarrheic calves [23,24], the following criteria were defined for initiation of antimicrobial therapy:

presence of a local infection (e.g. pneumonia, navel infection)

fever (> 39.5°C) or marked hypothermia (≤ 37°C)

hyperemia of the mucous membranes and/or congestion of the episcleral vessels

recumbency/inability to stand in calves ≤ 5 days of age.

If at least one of these criteria was met, calves received a preparation of amoxicillin at a dose of 10 mg/kg intravenously (Amoxisel®, Selectavet, Germany). Follow-up treatments were performed using a long-term formulation of amoxicillin (Hostamox® LA, 15 mg/kg, SC, Intervet Germany GmbH).

### Expected treatment failure

During the 24 hour study-period the investigator (FMT) was blinded to all laboratory findings. Two clinicians (AL and MF) assessed the laboratory parameters and calculated the expected correction of base excess (BE) by using the following formulas:

(3) $$\begin{array}{r} \text{theoretical change in BE}\;=\;\text{mmol}\;{\text{NaHCO}}_{3}\;\text{provided}\\ \;*\;\text{body mass}\;{\left(\text{kg}\right)}^{-1}\;*\;\text{factor}\;{\left(\text{l}/\text{kg}\right)}^{-1} \end{array}$$

(4) expected BE=actual BE+theoretical change in BE

A factor of 0.7 l/kg was used in formula (3) based on the results of a study by Lorenz and Vogt [25]. Calves with expected base excess values below −10 mmol/l were defined as treatment failures and were treated alternatively with the required amounts of sodium bicarbonate as determined above and infusion volumes estimated on the basis of clinical dehydration. These calves were not included in the analysis of data obtained after 24 hours but were used to optimize the assessed treatment protocol.

### Feeding

Calves were offered whole milk in a total volume of about 13% of body mass divided into three meals. One liter of a standardized oral rehydration solution was additionally offered three times within the 24 hours period between milk feedings. Some calves that were only treated orally received an additional liter of ORS on admission to test the intake of the solution. The oral rehydration solution contained 4 g NaCl, 20 g dextrose, 3 g KHCO_3_, and 3 g sodium propionate per liter. The calculated effective strong ion difference (SID) of this homemade solution is 61 mEq/l. Fresh water, hay and calf starter were offered ad libitum.

### Assessment of therapeutic success

Improvement of clinical and laboratory parameters was evaluated. Therapy of acidosis was considered to be successful if base excess values ranged between −5 and +5 mmol/l at the end of the 24 h study-period. A computer-assisted model was created in order to determine, and especially improve, the theoretical success of the assessed treatment regime. This approach was additionally used to analyze and compare the theoretical outcome of another previously published protocol [12]. The amount of sodium bicarbonate (NaHCO_3_) needed theoretically to correct acidosis was calculated for each calf after rearranging formula 3 as follows:

(5) $${\text{NaHCO}}_{3}\;\left(\text{mmol}\right)=\text{body mass}\;\left(\text{kg}\right)\;*\;\text{base deficit}\;\left(\text{mmol}/\text{l}\right)*\;0.7\;\left(\text{l}/\text{kg}\right)$$

Negative values in calves with alkalosis (due to a negative base deficit) were allowed. The calculated amount of sodium bicarbonate was then subtracted from the provided amount, which included the recorded intake of buffers from ORS.

### Statistical analysis

Results were analyzed statistically using SPSS 18.0 for Windows (IBM, New York, USA). Values of p < 0.05 were considered significant. Spearman’s coefficients of correlation were calculated in order to determine associations between parameters. Normal distribution was assessed visually using box-and-whisker plots and QQ plots. Since most of the data were not normally distributed, data are given as medians and the corresponding 25-/75-quartiles or minimum and maximum values. Mann–Whitney U-tests were used for comparisons of continuous parameters between groups. Differences between the five defined treatment groups were assessed using a Kruskal-Wallis test. For the subsequent pair-wise comparisons the Mann–Whitney-U-test was used as well. In this case the level of significance was adjusted using the Bonferroni method (p < 0.005). The initial and final values of investigated parameters were compared using paired Wilcoxon tests. The chi-squared test was performed to test for differences of categorical parameters between groups.

## Results

### Basal conditions, treatment and grouping of calves

The number of calves of each performed treatment procedure are given in Figure 1. The 121 calves were assigned to one of five treatment groups based on the provided infusion volumes and amounts of sodium bicarbonate as defined by the decision tree:

Group I: oral rehydration (n = 20)

Group II: constant drip infusion containing 250 mmol NaHCO_3_ (n = 47)

Group III: short-term infusion containing 500 mmol (n = 9) or 750 mmol NaHCO_3_ (n = 1)

Group IV: constant drip infusion containing 500 mmol NaHCO_3_ (n = 34)

Group V: constant drip infusion containing 750 mmol NaHCO_3_ (n = 10).

Calves that received sodium bicarbonate as short-term infusions were summarized to one treatment group for statistical reasons.

Antimicrobial therapy was deemed necessary in 66 calves (54.5%). Dextrose was added to infusion fluids in 20 cases. Due to severe ruminal acidosis milk and ORS were withheld in two of these calves. In the remaining 18 cases, 12 calves received a dextrose solution for reasons of hypothermia (≤ 37°C), 3 for reasons of a history of prolonged malnutrition, and 3 for reasons of suspected septicemia. The distribution of calves treated with dextrose and antibiotics among the five groups is given in Table 2.

**Table 2:** Proportion of calves in the five defined treatment groups that received dextrose and/or antibiotics

	**Group I**	**Group II**	**Group III**	**Group IV**	**Group V**
Dextrose	0/20	5/47	0/10	10/34	5/10
Antibiotics	3/20	27/47	4/10	22/34	10/10

Transfusion of 1 liter of whole blood was additionally performed in two three-day old calves of treatment group II because of distinct hypogammaglobulinemia as defined by PARISH et al. [26] (activity of gamma glutamyltransferase < 90 U/l).

The medians and 25-/75-quartiles for age (days) and body mass (kg) of the calves were 9.0 (6.0/12.5) and 43.0 (39.0/48.6), respectively. Statistically significant differences for body mass were only determined between treatment groups I and II (p = 0.004). The medians and 25-/75-quartiles were:

Group I: 47.1 (42.4/51.2) kg

Group II: 41.0 (37.0/46.3) kg

Group III: 47.3 (41.3/58.6) kg

Group IV: 43.9 (39.8/50.1) kg

Group V: 41.5 (35.8/45.0) kg.

### Expected treatment failure

**Figure 2:**
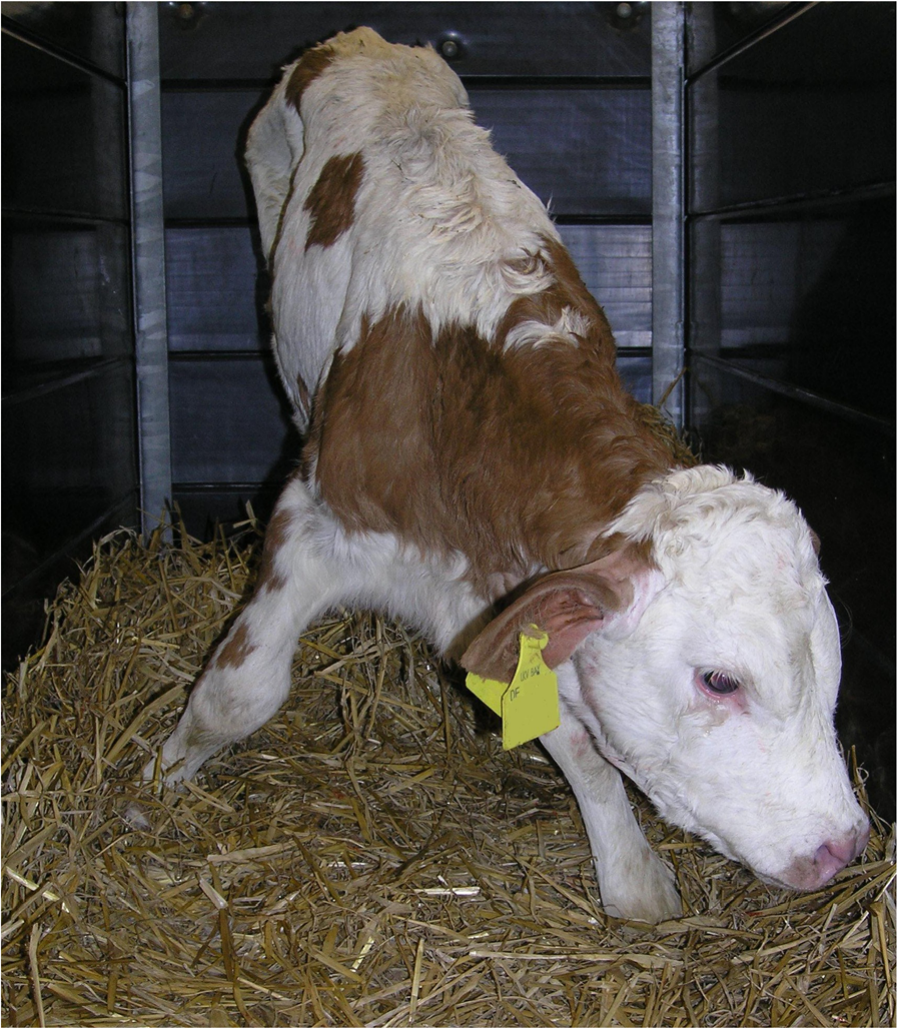
**Marked D-lactic acidosis in a diarrheic calf with expected treatment failure.** This calf was able to stand after being lifted but was not able to correct his position if pushed (posture score 5). Laboratory analysis revealed blood base excess of −28 mmol/l and D-lactate concentration of 12.4 mmol/l. This calf would have been underdosed with the intended amount of 500 mmol of sodium bicarbonate which would have an expected buffer capacity of 16.2 mmol/l (body weight 44 kg).

Five calves of treatment group IV had to be treated alternatively for reasons of expected treatment failure. All these calves were able to stand (albeit insecurely) in spite of a marked D-lactic acidosis with base excess values ranging from −24.3 to −28.0 mmol/l (median: −25.2 mmol/l). Thus metabolic acidosis would have been undercorrected with the provided amounts of 500 mmol sodium bicarbonate, which was intended for calves that stood insecurely. Additionally, undercorrection of metabolic acidosis was also caused by comparatively high body masses of these patients, which ranged from 44 to 56 kg (median: 53 kg). All these calves showed the same clinical picture on admission examination (Figure 2). All calves had to be helped to rise and were not able to correct position if pushed. They had an impaired palpebral reflex (delayed or absent) and responded only to painful stimuli (e.g. venipuncture).

### Clinical variables

Clinical signs of 116 calves that were treated with the help of the decision tree improved significantly during the investigation period (Table 1). Despite this fact, after 24 hours of therapy 52 calves (45%) had only a weak suckling reflex, whereas 19 calves had none. However, at the last milk feeding before the end of the study period, the offered milk volume was suckled entirely in 78 calves (67%). At least half of the offered milk volume was consumed by 22 calves (19%), while in the remaining 16 calves (14%) the milk intake was less satisfying (less than 50% of the offered milk volume).

Changes of clinical scores and the severity of enophthalmos in each treatment group are presented in Table 3. After 24 hours of therapy, statistically significant differences of clinical scores were only found for posture/ability to stand between treatment groups I and IV (p = 0.003) and for the palpebral reflex between treatment group II and III (p < 0.001) and II and V (p = 0.001), respectively.

**Table 3:** Clinical scores and severity of enophthalmos in different treatment groups on admission and 24 hours after the initiation of therapy

		**Group I:**	**Group II:**	**Group III:**	**Group IV:**	**Group V:**
		**Oral rehydration**	**Constant drip infusion**	**Short-term infusion**	**Constant drip infusion**	**Constant drip infusion**
			**250 mmol NaHCO**_**3**_	**500/750 mmol NaHCO**_**3**_	**500 mmol NaHCO**_**3**_	**750 mmol NaHCO**_**3**_
		**n = 20**	**n = 47**	**n = 10**	**n = 34/29 (0 h/24 h)**	**n = 10**
**Posture (score 1–7)**	0 h	2 (1–3)^NC^	2 (1–3)^NC^	4.5 (4–6)^NC^	5 (4–5)^NC^	6 (6–7)^NC^
	24 h	2 (1–2)^IV^	**2** (1–3)	**2** (1–2)	**2** (2–4)^I^	**2** (2–3)
**Behavior (score 1–5)**	0 h	1 (1–3)^NC^	2 (1–3)^NC^	3 (2–4)^NC^	3 (3–4)^NC^	4 (3–5)^NC^
	24 h	1 (1–3)	**2** (1–2)	**2** (1–3)	**2** (1–3)	**2** (1–3)
**Suckling reflex (score 1–3)**	0 h	1 (1–2)^NC^	2 (1–3)^NC^	2 (1–3)^NC^	3 (1–3)^NC^	3 (2–3)^NC^
	24 h	**2** (1–3)	2 (1–3)	2 (1–2)	**2** (1–3)	**2** (1–3)
**Palpebral reflex (score 1–4)**	0 h	1 (1–2)^NC^	1 (1–3)^NC^	3 (2–4)^NC^	2.5 (1–4)^NC^	3.5 (1–4)^NC^
	24 h	**1** (1–2)	**1** (1–2)^III,V^	**2** (1–3)^II^	**1** (1–2)	**1** (1–2)^II^
**Degree of enophthalmos (score 1–4)**	0 h	1 (1–1)^NC^	2 (1–4)^NC^	1 (1–1)^NC^	3 (1–4)^NC^	3 (2–4)^NC^
	24 h	1 (1–3)	**1** (1–3)	1 (1–1)	**1** (1–3)	**1** (1–2)
**Degree of skin tenting (score 1–4)**	0 h	2 (1–3)^NC^	3 (1–4)^NC^	3 (1–3)^NC^	4 (2–4)^NC^	4 (3–4)^NC^
	24 h	2 (1–4)	**2** (1–4)	2 (1–3)	**2** (1–3)	**2.5** (1–4)
**Severity of enophthalmos (mm)**	0 h	1 (0–3)^NC^	4 (1–10)^NC^	2 (1–3)^NC^	6 (3–12)^NC^	6.5 (4–11)^NC^
	24 h	1 (0–6)	**1** (1–6)	**1** (1–2)	**1** (0–6)	**2** (0–4)

Values are given as medians and corresponding ranges (minimum - maximum). Bold median values indicate statistically significant changes during the investigation period. Five out of 34 calves of treatment group IV had to be treated alternatively for reasons of expected treatment failure. Comparisons between times of examination were performed for the remaining 29 calves of this group. Superscripts indicate number of groups with significantly different values after pairwise comparisons. Since grouping of calves was based on clinical findings and most of the given parameters are closely correlated with each other the initial values were not compared (indicated by ^NC^).

### Laboratory variables

**Figure 3:**
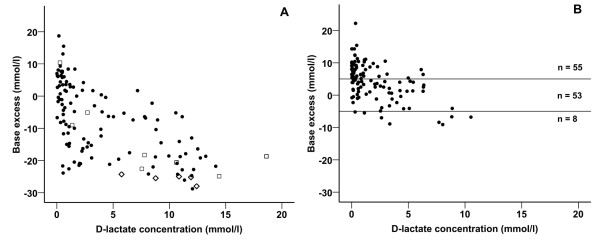
**Base excess values and D-lactate concentrations of calves at times of examination.A**: Blood base excess and D-lactate concentrations in 121 calves with neonatal diarrhea on admission. Values of five calves that had to be treated alternatively for reasons of expected treatment failure are indicated by ◊. Values of calves in which metabolic acidosis was not corrected successfully are indicated by □. **B**: Outcome of therapy regarding blood base excess and D-lactate concentrations in 116 diarrheic calves that were treated according to the developed decision tree. Therapy was considered to be successful, if base excess values ranged between −5 and +5 mmol/l at the end of the 24 h study period. After 24 hours eight calves still showed base excess values below −5 mmol/l, whereas a metabolic alkalosis (base excess above +5 mmol/l) was present in 55 calves.

Blood base excess and D-lactate concentrations of calves on admission are presented in Figure 3A. Table 4 shows changes of selected laboratory variables in different treatment groups of 116 calves that were treated using the decision tree. No significant difference in base excess values on admission could be detected between calves of groups III and IV on the one hand and calves of group V on the other hand. D-lactate concentrations were most massively increased in calves of group III (impairment of ability to stand, no enophthalmos). After the 24 hour-study period values for base excess, anion gap, creatinine and total protein were not significantly different between treatment groups. The intended amounts of sodium bicarbonate and its equivalents (which were based on simple clinical findings) correlated significantly (p < 0.001) with base excess (r = −0.74), D-lactate (r = 0.55) and anion gap (r = 0.77) on admission.

**Table 4:** Blood concentrations of selected laboratory parameters in different treatment groups on admission and at the end of the study period

		**Group I:**	**Group II:**	**Group III:**	**Group IV:**	**Group V:**
		**Oral rehydration**	**Constant drip infusion**	**Short-term infusion**	**Constant drip infusion**	**Constant drip infusion**
			**250 mmol NaHCO**_**3**_	**500/750 mmol NaHCO**_**3**_	**500 mmol NaHCO**_**3**_	**750 mmol NaHCO**_**3**_
		**n = 20**	**n = 47**	**n = 10**	**n = 34/29 (0 h/24 h)**	**n = 10**
**Base Excess (mmol/l)**	0 h	2.8 (−5.3/7.4)^III, IV, V^	0.0 (−8.8/4.2)^III, IV, V^	−17.2 (−19.9/-9.7)^I, II^	−18.9 (−22.7/-14.5) ^I, II^	−22.9 (−25.1/-19.0) ^I, II^
	24 h	4.5 (0.8/8.5)	**5.3** (1.8/7.9)	**3.1** (−4.1/5.6)	**2.1** (−1.2/8.0)	**7.8** (3.7/11.2)
**D-Lactate (mmol/l)**	0 h	0.9 (0.2/5.6)^III, IV^	0.8 (0.5/2.4)^III, IV, V^	11.7 (8.7/12.9)^I, II, IV^	7.0 (1.9/10.8)^I, II, III^	6.4 (2.1/12.0)^II^
	24 h	0.5 (0.2/2.6)^III^	**0.3** (0.1/0.9)^III, IV, V^	**4.5** (2.7/6.8)^I, II^	**1.3** (0.3/4.6)^II^	**1.5** (0.7/4.6)^II^
**L-Lactate (mmol/l)**	0 h	1.1 (0.7/2.0)^II^	2.7 (1.4/4.1)^I, III^	0.6 (0.4/0.6)^II, IV, V^	2.7 (0.9/5.9)^III^	2.6 (0.8/6.0)^III^
	24 h	1.2 (0.9/2.2)	**1.5** (1.1/2.1)^III^	**0.9** (0.7/1.2)^II, V^	**1.4** (1.0/2.1)	1.6 (1.0/3.0)^III^
**Total protein (g/l)**	0 h	53.6 (46.5/56.8)^IV, V^	58.2 (51.9/66.0)^V^	50.9 (48.7/59.8)^IV, V^	64.5 (57.1/74.8)^I, III^	72.4 (63.4/80.4)^I, II, III^
	24 h	**50.8** (43.8/55.0)	**49.6** (43.6/53.8)	**46.2** (43.9/51.4)	**48.0** (43.0/54.5)	**47.4** (43.2/58.6)
**Urea (mmol/l)**	0 h	6.0 (3.3/8.3)^II, IV, V^	13.0 (6.7/20.4)^I, IV; V^	8.4 (6.9/10.9)^IV, V^	17.9 (15.2/26.9)^I, II, III^	29.2 (19.6/34.1)^I, II, III^
	24 h	4.7 (3.0/7.7)^IV, V^	**5.0** (3.8/9.4)	**4.7** (3.7/5.7)^IV, V^	**8.3** (5.2/13.7)^I, III^	**11.9** (9.5/19.2)^I, III^
**Creatinine (μmol/l)**	0 h	100.6 (76.0/124.6)^IV, V^	130.8 (87.7/271.9)^IV, V^	95.1 (73.1/134.4)^IV, V^	262.9 (148.4/433.5)^I, II, III^	411.1 (188.3/623.3)^I, II, III^
	24 h	81.3 (72.6/128.9)	**80.7** (63.6/120.2)	**72.1** (66.5/88.2)	**98.1** (70.4/207.5)	**112.3** (81.3/252.4)
**Potassium (mmol/l)**	0 h	4.5 (4.1/4.9)^II, IV, V^	5.1 (4.5/6.0)^I, III, V^	4.1 (3.9/4.5)^II, IV, V^	6.0 (4.4/7.3)^I, III^	8.1 (5.6/8.5)^I, II, III^
	24 h	**4.7** (4.4/5.1)^IV^	**4.6** (4.3/5.0)	4.4 (3.5/4.8)	**4.3** (3.8/4.5)^I^	**4.5** (3.5/5.0)
**Sodium (mmol/l)**	0 h	134.0 (129.9/135.8)	133.2 (129.3/137.2)	136.0 (133.9/137.6)	135.4 (130.2/143.3)	128.4 (115.7/144.1)
	24 h	133.2 (131.6/137.9)	**135.2** (133.1/137.8)	133.0 (131.9/136.4)	137.1 (134.4/143.8)	**138.9** (135.3/145.4)
**Anion gap (mEq/l)**	0 h	12.3 (8.7/17.1)^III, IV, V^	16.6 (12.8/22.7)^III, IV, V^	24.2 (21.4/28.2)^I, II, IV, V^	28.2 (26.4/34.6)^I, II, III^	31.9 (29.8/36.9)^I, II, III^
	24 h	**11.4** (7.4/14.0)	**9.7** (7.2/12.3)	**11.5** (9.1/16.5)	**12.5** (9.6/14.8)	**12.6** (8.5/18.0)

Values are given as medians and 25-/75-quartiles. Superscripts indicate number of groups with significantly different values after pairwise comparisons. Bold median values indicate statistically significant changes during the investigation period. Five out of 34 calves of treatment group IV had to be treated alternatively for reasons of expected treatment failure. Comparisons between times of examination were performed for the remaining 29 calves of this group.

### Outcome of therapy

**Figure 4:**
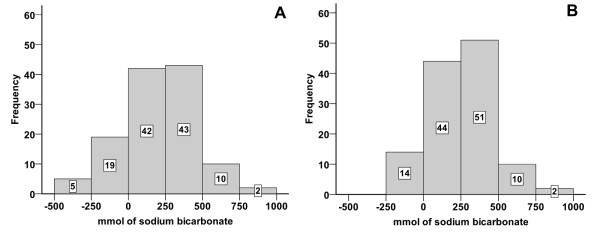
**Theoretical outcome of therapy of metabolic acidosis in 121 diarrheic calves using a computer-assisted analysis.** Positive values indicate calves that are theoretically overdosed, whereas negative values indicate calves that were theoretically underdosed (calculated with formula 3). The buffer content of the recorded intake of ORS was included in this computational analysis. **A**: Theoretical outcome of therapy using the evaluated decision tree. Metabolic acidosis was theoretically undercorrected by considerable amounts of sodium bicarbonate (> 250 mmol) in five calves. In these five calves treatment failure was expected. **B**: Theoretical outcome of therapy using the decision tree in the modified version. 14 calves would still have been underdosed by small amounts of sodium bicarbonate (range: 0.3 - 155 mmol).

Results of the theoretical outcome of the treatment regime, with positive values indicating overdosing and negative values indicating underdosing by the provided amounts of sodium bicarbonate are presented in Figure 4A. This computational analysis revealed that five calves were underdosed with quantities of more than 250 mmol sodium bicarbonate. These calves were identified as the five expected treatment failures mentioned above. Results of the real outcome of therapy regarding blood base excess and D-lactate concentrations at the end of the investigation period are presented in Figure 3B. 55 calves (45.5%) were overdosed with the provided amounts of sodium bicarbonate. Eight calves still showed base excess levels below −5 mmol/l. As mentioned above, five calves had to be treated alternatively for reasons of expected treatment failure. Thus metabolic acidosis could not be treated adequately in 13 calves (10.7%). Body masses of calves were not significantly different between successfully treated calves and all calves that were underdosed with the provided amounts of sodium bicarbonate (p = 0.10).

As indicated by the presence of persistent enophthalmos (visible gap between the eyeball and *caruncula lacrimalis*) dehydration was not corrected in 17 calves. Table 5 shows the proportion of still dehydrated calves at the end of the study period depending on the severity of enophthalmos (mm) on admission. A subset of 14 out of 28 calves with a measured enophthalmos ≥ 7 mm was not rehydrated successfully in the first 24 hours.

**Table 5:** Number of still dehydrated calves at the end of the study period depending on the severity of enophthalmos (mm) on admission

**Enophthalmos on admission (mm)**	**Calves**	Not successfully rehydrated*
	**n**	**n**
5	10	0
6	14	1
7	10	3
8	5	1
9	5	2
≥ 10	8	8

* visible gap between the eyeball and caruncula lacrimalis (corresponding to a measured eye ball recession of at least 3–4 mm).

### Iatrogenic alkalosis

After the investigation period, a metabolic alkalosis (base excess levels > +5 mmol/l) was present in 55 calves. However, 24 of these overdosed calves had already positive base excess values on admission. This was attributed to pretreatment in 11 patients, which had initial base excess values ranging from 1.8 to 18.7 mmol/l. All clinical and laboratory parameters investigated were compared between successfully treated calves (BE values ranging from −5 to +5 mmol/l) and calves that were overdosed with the provided amounts of sodium bicarbonate. Selected parameters are listed in Table 6. Calves with metabolic alkalosis were younger and exhibited significantly higher base excess values, higher L-lactate and lower D-lactate concentrations before treatment. Body masses of calves did not differ significantly between groups. Clinical findings including the suckling behavior at the last milk feeding before the end of the study-period revealed no detectable adverse effects of this metabolic disturbance (Table 7).

**Table 6:** Age, body mass and selected laboratory parameters in relation to the outcome of therapy

**Parameter**	**Successfully treated**	**Overdosed calves**	**p value**
	**(BE −5 to +5 mmol/l)**	**(BE > +5 mmol/l)**	
	**n = 53**	**n = 55**	
**On admission**			
Age (days)	10.0 (6.5/14.0)	8.0 (5.0/11.0)	0.046
Body mass (kg)	43.0 (38.8/48.7)	42.2 (38.2/47.6)	0.537
Base excess (mmol/l)	−14.5 (−19.4/-5.1)	−1.0 (−10.4/5.8)	< 0.001
HCO_3_^-^ (mmol/l)	15.8 (9.9/22.2)	25.5 (18.9/31.3)	< 0.001
Venous PCO_2_ (mm Hg)	47.3 (37.6/56.1)	56.4 (48.5/61.2)	< 0.001
D-lactate (mmol/l)	5.0 (1.1/10.3)	0.8 (0.4/3.1)	< 0.001
L-lactate (mmol/l)	1.3 (0.7/3.3)	2.7 (1.3/5.2)	0.002
Anion gap (mEq/l)	24.4 (20.4/27.9)	16.2 (12.0/27.5)	0.008
Sodium (mmol/l)	136.3 (130.8/140.3)	131.6 (129.1/135.4)	0.002
Chloride (mmol/l)	101.0 (95.0/107.0)	95.0 (88.0/98.0)	< 0.001
Potassium (mmol/l)	4.8 (4.3/6.0)	5.2 (4.5/6.8)	0.153
Ionized Calcium (mmol/l)	1.3 (1.2/1.3)	1.2 (1.1/1.3)	0.004
**After 24 hours**			
Base excess (mmol/l)	1.5 (−0.8/3.8)	8.0 (6.4/10.3)	< 0.001
HCO_3_^-^ (mmol/l)	27.6 (25.3/29.7)	34.4 (32.3/36.1)	< 0.001
Venous PCO_2_ (mm Hg)	53.2 (48.9/57.4)	59.9 (55.5/63.7)	< 0.001
D-lactate (mmol/l)	2.1 (0.5/4.1)	0.4 (0.1/1.1)	< 0.001
L-lactate (mmol/l)	1.3 (1.0/2.2)	1.5 (1.1/2.1)	0.747
Anion gap (mEq/l)	12.7 (10.0/14.8)	9.0 (6.6/11.6)	< 0.001
Sodium (mmol/l)	135.0 (133.0/140.0)	135.9 (132.9/138.3)	0.768
Chloride (mmol/l)	101.0 (99.0/106.0)	97.0 (95.0/99.0)	< 0.001
Potassium (mmol/l)	4.5 (4.2/4.9)	4.6 (4.2/4.9)	0.507
Ionized Calcium (mmol/l)	1.2 (1.1/1.3)	1.2 (1.1/1.2)	0.283

Values are given as medians and 25-/75-quartiles.

**Table 7:** Clinical parameters and the suckling behavior at the end of the study period in relation to the outcome of therapy

**Clinical Scores**	**Successfully treated**	**Overdosed calves**	**p value**
	**(BE −5 to +5 mmol/l)**	**(BE > +5 mmol/l)**	
	**n**	**n**	
**Posture**			
1	8	4	
2	43	49	0.430
3	2	2	
**Behavior**			
1	24	24	
2	27	31	0.326
3	2	0	
**Suckling reflex**			
1	23	22	
2	25	20	0.129
3	5	13	
**Palpebral reflex**			
1	43	52	0.032
2	10	3	
**Degree of enophthalmos**			
1	44	48	
2	7	6	0.761
3	2	1	
**Degree of skin tenting**			
1	20	15	
2	17	25	0.469
3	14	14	
4	2	1	
Suckling behavior*			
100%	37	36	
50 - 99%	11	10	0.558
< 50%	5	9	

* regarding the intake of the offered milk volume at the last feeding before the end of the study period.

### Predominant laboratory and clinical findings in calves with undercorrection of metabolic acidosis on admission

Since bicarbonate requirements were mainly based on alterations in posture/ability to stand, the success of therapy was evaluated with regard to categories of this parameter. Table 8 shows the results of this analysis. Metabolic acidosis was most often not successfully corrected in patients that stood insecurely and were not able to correct position if pushed (p = 0.007). Nine of the 13 underdosed calves fell into that category on admission (including the five calves with expected treatment failure). All these nine calves suffered from marked D-lactic acidosis with base excess values ranging from −18.7 to −28 mmol/l (Median: −24.9 mmol/l) and D-lactate concentrations ranging from 5.8 to 18.6 mmol/l (Median: 10.9 mmol/l). Impairment of the palpebral reflex was additionally recognized in all nine patients on admission. Therefore it was analyzed whether calves that were barely able to stand (unable to correct position if pushed) require higher doses of sodium bicarbonate in general, or whether these buffer requirements depend on the quality of the palpebral reflex. In all 29 calves with this clinical sign, the outcome of therapy of metabolic acidosis depended strictly on the degree of loss of the palpebral reflex (Table 9). In all calves exhibiting a prompt palpebral reflex, base excess values were positive, whereas in calves with a delayed or absent palpebral reflex, base excess values were still predominantly negative after 24 hours of therapy.

**Table 8:** Success of therapy (correction of acidosis) in 121 diarrheic calves depending on alterations in posture/ability to stand on admission

**Posture/ability to stand on admission**	**Successfully treated**	**Not successfully treated**
	**n**	**n**
Standing up by itself	6	1
Standing up after encouragement	51	2
Standing securely after lifting	7	0
Standing insecurely, able to correct position if pushed	13	1
Standing insecurely, not able to correct position if pushed	20	9
Sternal recumbency	7	0
Lateral recumbency	4	0

Differences between groups are statistically significant (p = 0.007).

**Table 9:** Selected parameters depending on alterations of the palpebral reflex in 29 calves that stood insecurely and were not able to correct position if pushed

**Parameter**	**Palpebral reflex**	**Palpebral reflex**	**p value**
	**prompt**	**delayed or absent**	
	**n = 9**	**n = 20**	
Base excess^*^ (mmol/l) after 24 h	6.8 (2.7/8.8)	−4.2 (−10.4/-0.6)	< 0.001
Base excess (mmol/l) on admission	−17.1 (−20.4/-13.0)	−22.3 (−24.8/-18.7)	0.007
D-lactate (mmol/l) on admission	2.2 (1.3/3.9)	10.8 (8.9/12.3)	< 0.001
Potassium (mmol/l) on admission	7.5 (7.0/8.0)	4.7 (3.9/6.0)	< 0.001
Enophthalmos (mm)	6.0 (5.0/10.5)	4.0 (3.3/6.8)	0.039
Body mass (kg)	41.8 (38.3/46.2)	45.5 (39.4/52.4)	0.365

Values are given as medians and 25-/75-quartiles.

*Values for calves with expected treatment failure were calculated using formula 3 and 4.

### Therapy after the study period

Since most calves were still diarrheic at the end of the study period and acidosis and/or dehydration was not corrected in some of the patients, further treatment based on the actual acid–base status, clinical dehydration and the assumed on-going losses of fluids and buffer substances was required. After a median duration of 9 days of hospitalization 95% of the 121 calves (n = 115, including the five defined treatment failures) were discharged in a healthy state as defined by a normal consistency of the feces for at least two days.

## Discussion

The aim of the present study was to investigate whether a decision tree developed on the basis of a retrospective analysis of data [9] can be used successfully to treat metabolic acidosis in diarrheic calves without expensive laboratory equipments. Since the ultimate goal of the study was to create recommendations for veterinary practitioners the decision tree uses only very basic clinical signs for the prediction of the required amounts of buffer substances. However, the fact that both, the retrospective and the present study were conducted on calves in a hospital setting created some problems for the study design, as well as the transferability of the results into a field practice situation. In practice the obvious measure of success of treatment is the clinical recovery of the calf, especially judged by the improvement of milk intake. Under the situation of a hospital the calves are exposed to many additional stressors such as transport, change of environment and personnel as well as changes in feeding system and type of milk. As a result changes in feed intake are most likely not comparable to calves that were treated under farm conditions. An example for the influence of hospital related management factors on clinical signs is the lack or weakness of the suckling reflex in many calves after 24 hours even though many clinical examinations were performed shortly after the feeding of calves and most calves drank all or parts of the offered milk ration. For this reason a base excess value of −5 mmol/l was chosen as a cut point for treatment success. Previous studies have shown, that this value roughly correlates to the point where calves still show an impairment of ability to stand [25,27], and where D-lactate concentrations of calves are most likely still above 6 mmol/l [27], which in itself has been described as a threshold for clinical signs in an experimental study [28]. Considering that there is a well proven relationship between D-lactate concentrations and clinical signs in diarrheic calves [7,8] it can therefore be expected that the current study will provide results that are valuable not only in clinical settings but also in field practice.

Similar median base excess values in different treatment groups at the end of the investigation period and the similar clinical picture of most of the few calves that were not successfully treated confirm the success of our blinded study. The coefficients of correlation between the intended amounts of sodium bicarbonate (which are based on basic clinical signs) on the one hand, and base excess values and anion gap on the other, were highly significant. These findings do not confirm studies which questioned the relation between clinical signs and degree of acidosis [29,30]. Compared with the anion gap, a lower correlation coefficient existed between the provided amounts of sodium bicarbonate and D-lactate concentrations. This may be due to the fact that both D- and L-lactate contribute to elevation of the anion gap in diarrheic calves [4].

The clinical status of 116 calves that were treated with the aid of the decision tree improved significantly during the study period of 24 hours. Additionally, almost all clinical findings did not differ significantly between treatment groups at the end of the investigation period. Statistically significant differences were found for posture between treatment groups I and IV, which can be explained by the fact that metabolic acidosis was not completely corrected in 5 calves of treatment group IV. After 24 hours, calves of treatment group III had significantly higher scores corresponding to the degree of loss of the palpebral reflex than calves of treatment group II. This finding can be explained by still elevated D-lactate concentrations in some calves of treatment group III, which are closely correlated to disturbances of the palpebral reflex [7,8,17]. Since no low values for D-lactate could be determined in any of the calves of group III on admission, these patients represent the so called “acidosis without dehydration syndrome”. This clinical picture was first described in clinically non-dehydrated Charolais calves without significant diarrhea [31]. The authors characterized the syndrome by depression of the CNS, ataxia, recumbency and coma and assumed unknown organic or inorganic acid as the cause of the increase in anion gap detectable in these patients. This speculation was clarified by Schelcher et al. [32] who reported D-lactic acid as the cause of this syndrome of high anion gap acidosis.

The amount of sodium bicarbonate administered to the calves was determined with the aid of the decision tree without regarding individual body mass. We concluded that this circumstance had only minor influence on therapeutic success since body masses were not significantly different between successfully treated calves, on the one hand, and overdosed or underdosed calves, on the other hand. However, in some individual cases body mass had some importance. This was especially true for most calves with expected treatment failure.

The five calves with expected treatment failure were treated differently from the assessed protocol since a previous study [25] has shown that not only prior to initial treatment, but also later in the course of therapy, the clinical signs are correlated to a still existing/reoccurring acidosis. We cannot rule out that those calves would have recovered in spite of underdosing of bicarbonate, but in the light of this study [25] and daily routine at our clinic it was deemed very unlikely. This assumption is a weakness of the study, but it was decided for the benefit of the patients. For the same reason transfusion of 1 liter of whole blood was also deemed necessary in two three-days old calves of group II because of suspected failure of passive transfer of colostral immunoglobulins. This therapeutic procedure may have had an influence on various laboratory variables in these patients. But this effect should be negligible in view of the large number of calves in this group. The serum total protein concentrations of calves 24 h after the initiation of therapy may imply that hypogammaglobulinemia was a problem in the majority of the calves included in the study. However, it is likely that a catabolic state due to low milk intake before referral to the clinic may have further contributed to this finding in many cases.

Outcome of therapy confirms the results of previous analysis [9,17], which demonstrated that the provided dosages of sodium bicarbonate are more likely to overdose than to underdose calves with diarrhea and metabolic acidosis. However, no detrimental effects of metabolic alkalosis were observed in our calves regarding the investigated parameters, leading to the conclusion that moderate overdosing with sodium bicarbonate is more desirable than underdosing. Several factors may have contributed to the metabolic alkalosis in 45.5% of the calves after treatment:

Patients that are admitted to a university teaching hospital are usually preselected. In some cases, calves had been pre-treated with large amounts of sodium bicarbonate but not enough volume of infusion before referral. Thereby they were still clinically dehydrated upon arrival and received infusions with additional sodium bicarbonate.

Intake of ORS was tested in calves that were intended for oral rehydration as sole therapy. Ten of these patients refused to drink ORS immediately following examination on admission. Therefore they received a constant drip infusion with 250 mmol of sodium bicarbonate. It is likely that the stress involved with admission in the hospital also played a significant role here. In general, it has to be assumed that the first branch of the decision tree plays a minor role in practice since a practitioner is unlikely to be called to treat a diarrheic calf that is clinically unaffected.

Overdosed calves were significantly younger and had significantly lower D-lactate concentrations than successfully treated patients. Naylor [33] has shown that diarrheic calves during their first week of life are less acidemic than older calves with similar clinical signs. This finding was included in several protocols for determining bicarbonate requirements for diarrheic calves under field conditions [11-13]. However, in our study, age had no clear influence on the accuracy of the estimation of the degree of acidosis based on alterations in posture/ability to stand and was therefore not considered to be of clinical relevance. Nevertheless, patients that suffered from diarrhea within their first week of life had significantly higher base excess values and significantly lower D-lactate concentrations than older calves [17]. Probably, metabolic acidosis was easier to correct (or overcorrect) in younger calves, since a significant correlation has been reported between D-lactate concentrations and the factor used in formula 3 [25].

Even though patients with incurable diseases were disregarded, calves with further health problems were not explicitly excluded in our study. Thus it cannot be completely ruled out that clinical signs in some calves were more pronounced due to concurrent problems (e.g. pneumonia or navel infection). Therefore, some calves may have received higher amounts of sodium bicarbonate than necessary.

The treatment protocol used in this study is based on 5 liter bags of isotonic saline and 8.4% hypertonic sodium bicarbonate solution, which was either diluted or used to spike the isotonic saline. It has been shown previously that slightly hypertonic sodium bicarbonate solutions can be used safely to achieve a faster correction of acidosis than with isotonic solutions [25,34]. We used 4.2% sodium bicarbonate solution as sole treatment in calves without clinically relevant dehydration, and 4.2% or 2.1% solution was given in dehydrated calves prior to the 5 liters of isotonic saline spiked with 250 ml of 8.4% sodium bicarbonate solution. The reason for the latter was to insure that correction of acidosis is achieved even in situations (under field conditions) where the entire determined infusion volume is not administered because of technical problems. It is obvious that the undiluted use of the commercially available 8.4% hypertonic sodium bicarbonate solution would greatly simplify the suggested treatment protocol. However, a recent review paper discussed the risks of the direct use of 8.4% sodium bicarbonate solution and cautioned against it in severely dehydrated calves that are unable to suckle as well as in cases of diarrheic patients with concurrent respiratory disease and/or hypernatremia [13]. To our knowledge the use of 8.4% sodium bicarbonate solution has been described in only two studies so far in calves with naturally acquired diarrhea. Koch and Kaske [35] compared the clinical efficacy of hypertonic saline and sodium bicarbonate solutions combined with the administration of an oral electrolyte solution. They reported good results and no major adverse reactions in 12 calves receiving an 8.4% sodium bicarbonate solution at a dosage of 10 ml/kg over 8 minutes. Coskun et al. [36] used the same solution at a low infusion speed of 10 ml/kg over 20 minutes and could also not observe any adverse signs. The use of 8.4% sodium bicarbonate solution as premedication followed by a constant drip infusion, as well as the combination of a fast infusion to capitalize on the resuscitative effect in combination with oral rehydration would be very beneficial in field practice. However, the accumulation of more clinical data would be desirable before a recommendation on the safe and successful use can be given.

For means of rehydration we used a volume of 5 liters of isotonic saline per calf, irrespective of the degree of dehydration to test the hypothesis that taking into account the oral fluid intake, all calves would be successfully rehydrated using this volume. However, our results indicate that the provided infusion volumes of 5.25 to 6.25 liters in total were too low for severely dehydrated calves. In particular, normal hydration was not completely restored in 50% of calves with an enophthalmos of ≥ 7 mm on admission. Therefore administration of 10 liters instead of 5 liters of isotonic saline (in consideration of commercially available packages) seems to be advisable for such patients when determining the treatment for a period of 24 hours. Especially in severely sick diarrheic calves which are in need of a constant drip infusion we recommend a re-evaluation of the calf after 24 hours in order to decide on the proper follow-up treatment by using the decision tree. Even though the oral route for further fluid therapy would be preferable, this may not always be possible, especially in weak calves with a low milk intake and high intestinal losses of fluids and buffer substances. A clinical study on the dynamics of D-lactate concentration during the course of therapy has also shown that calves with a marked D-lactic acidosis may need a repeated treatment with buffer substances for correction of acidosis (and D-lactatemia) and the associated clinical signs [25].

In 13 calves of the present study, metabolic acidosis was not corrected in a satisfactory manner. Nine of these calves showed the same clinical picture on initial examination. Several reasons were found in the remaining four isolated cases, which may explain undercorrection of metabolic acidosis. One calf that received ORS as sole treatment suffered from profound diarrhea (watery consistency of feces) and had a low intake of milk and ORS. Thus base excess decreased from +10.4 on admission to −5.2 mmol/l after 24 hours. In two other calves, development of ruminal acidosis due to ruminal drinking was recognized during the study period. At the end of the study period ruminal liquid had massively increased (> 1 liter), and contained large amounts of caseinate, and a pH of 5.4 was measured in both cases. Ruminal drinking is a frequently observed complication of neonatal diarrhea [37] and bacterial fermentation of substrates in the rumen and subsequent absorption of D-lactate can contribute to systemic metabolic acidosis in this situation [38]. No intake of ORS was recorded in a calf of treatment group II and metabolic acidosis was not corrected fully in this patient (base excess −5.5 mmol/l after 24 hours).

Clinical findings other than those used in the decision tree had to be identified in order to improve the success of the treatment protocol. Our analysis revealed that calves with barely maintained ability to stand (not able to correct position if pushed) require higher doses of sodium bicarbonate if there is evidence of a marked D-lactic acidosis indicated by delay or absence of the palpebral reflex. Two aspects may have contributed to this finding:

A previous clinical study reported that the risk of failure to correct acidosis increases with D-lactate concentrations [25].

In our investigation, many calves were able to stand despite base excess levels below −20 mmol/l. Most of these calves stood insecurely and were thereby underdosed with the provided amounts of 500 mmol sodium bicarbonate, which has a buffer capacity of approximately 15–20 mmol/l in calves of that size.

Adequate assessment and categorization of clinical parameters, especially attempts to lift the calves to their feet, may have contributed to the latter point. In addition, it may explain the lack of a significant difference in the base excess values between treatment groups III and IV, on the one hand, and the treatment group V, on the other hand. In the light of the finding that moderate alkalosis does not seem to have any detrimental effect it can be speculated that less accurate clinical examinations, without lifting of the calf when it was unable or unwilling to stand up, might have resulted in a smaller number of underdosed calves. However, since a clinical impact of higher degrees of alkalosis cannot be excluded on the basis of this study, and higher amounts of bicarbonate provided will also increase the costs of treatment it is still reasonable to avoid severe overdosing, as it would have occurred without the attempt to lift the calves e.g. in calves that stood securely after lifting. Even though an attempt to lift the calf to its feet may not always be feasible in ambulatory practice, it allows the veterinarian to perform a more detailed clinical examination and consequently more accurate estimation of the degree of acidosis, especially in calves where an inability to stand is expected.

**Figure 5:**
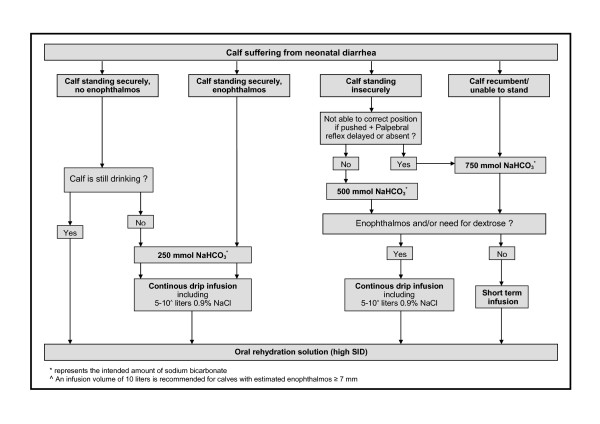
**Recommended version of the decision tree for treating metabolic acidosis in calves with neonatal diarrhea in bovine field practice.** Examination of the posture/ability to stand includes lifting of the animal if it is not able or willing to stand up. The term enophthalmos is defined as a visible gap between the eyeball and caruncula lacrimalis, which corresponds to a measured eyeball recession of at least 3–4 millimeters.

We used a computer-assisted analysis to test the theoretical outcome of the treatment regime, if calves showing distinct impairment of ability to stand (not able to correct position if forced) and a delay or absence of the palpebral reflex would have received an additional 250 mmol of sodium bicarbonate. For this analysis, it was assumed that calves with expected treatment failure had additionally received 3 liters of ORS. Figure 4B shows the result of this theoretical analysis (with positive values indicating calves that would have been overdosed and negative values indicating calves that would have been underdosed). A total of 14 calves would still have been underdosed with small amounts of sodium bicarbonate (< 250 mmol). The still missing amounts of sodium bicarbonate needed theoretically to correct acidosis (base excess 0.0 mmol/l) varied between 3 and 79 mmol in 13 of these 14 calves and appear to be negligible for the treatment of acidosis under field conditions. Figure 5 shows the decision tree in the recommended version which should substantially facilitate the treatment of diarrheic calves in bovine practice.

**Figure 6:**
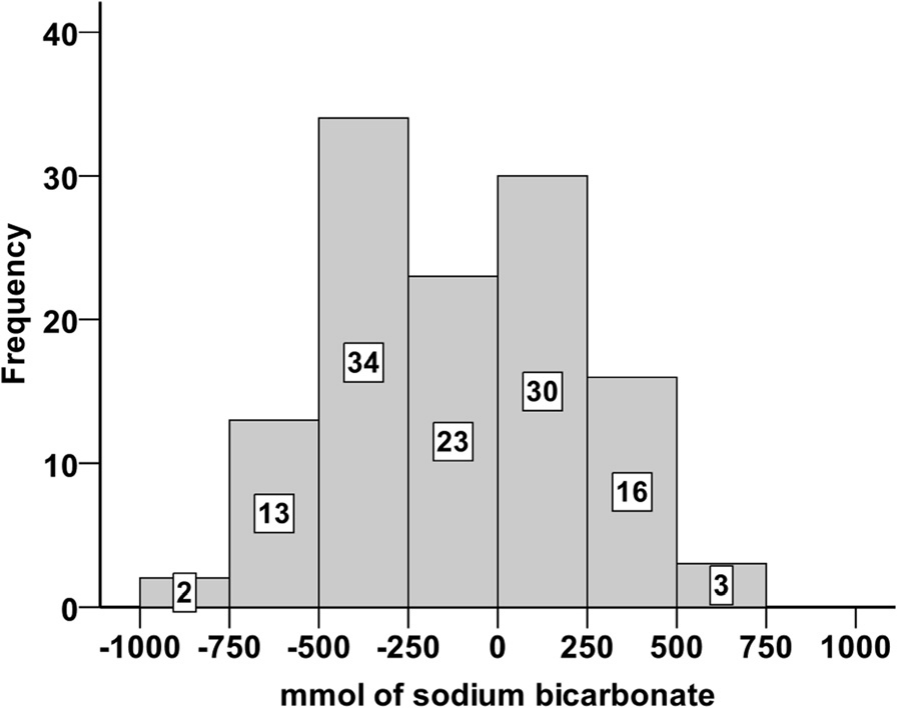
**Theoretical outcome of therapy of metabolic acidosis in 121 diarrheic calves based on previously published recommendations for the dosage of sodium bicarbonate **[12]. Positive values indicate calves that were theoretically overdosed, whereas negative values indicate calves that were theoretically underdosed (calculated with formula 3a). The intended amounts of sodium bicarbonate are based on posture/ability to stand, age and body mass of calves. For calves that would have received an oral rehydration solution, the buffer content of the recorded intake of ORS was used to calculate the theoretical success of therapy (an equivalent of 183 mmol of sodium bicarbonate was used for calves with expected treatment failure).

The outcome of the treatment protocol indicates that the dosages of sodium bicarbonate required for correction of metabolic acidosis in diarrheic calves are in many cases higher than those of previously published guidelines or protocols [12-14]. Figure 6 shows the theoretical outcome of a suggested protocol which used posture/ability to stand, age and the estimated body weight of calves as decision criteria for the determination of bicarbonate requirements [12]. Calves presented in sternal or lateral recumbency would have received infusions with dosages of sodium bicarbonate ranging from 150 to 600 mmol depending on body weight and age of the calf. Calves that were still able to stand would have received an oral rehydration solution as sole therapy. Using these recommendations, 49 calves (40.5%) would have been underdosed by considerable amounts (> 250 mmol) of sodium bicarbonate.

In the surveyed literature, maximum dosages of 500 to 620 mmol of sodium bicarbonate were generally regarded to be sufficient for intravenous correction of metabolic acidosis in calves with severe depression and sternal or lateral recumbency. Especially a wide variation of recommendations with dosages ranging from 75 to 500 mmol of sodium bicarbonate or its equivalents have been recommended for calves standing insecurely which may be explained by the fact that these protocols are based on practical experiences or on retrospective studies [12-14]. Retrospective studies usually lack standardization of clinical examination techniques and recording of clinical signs, which is not the case in this prospective study.

## Conclusions

This study evaluated the success and feasibility of a protocol for the treatment of diarrheic calves with emphasis on the required minimal amounts of sodium bicarbonate for sufficient correction of metabolic acidosis. We conclude that bicarbonate requirements can be estimated on the basis of posture/ability to stand and degree of dehydration as recommended by Lorenz and Lorch [9]. However, calves that stand insecurely and are not able to correct their position if pushed require higher doses of sodium bicarbonate than previously suggested, if there is clinical evidence of a marked D-lactic acidosis. Determining the degree of loss of the palpebral reflex, which had been reported to be a reliable clinical tool for diagnosing elevations of D-lactate concentrations [7,8], was identified as a useful decision criterion to provide an additional amount of sodium bicarbonate in those calves. This work shows the clinical relevance of the discovery that D-lactate is responsible for most of the clinical signs exhibited by neonatal diarrheic calves suffering from metabolic acidosis.

## Abbreviations

BE: Base excess; CNS: Central nervous system; ORS: Oral rehydration solution; SID: Strong ion difference.

## Competing interests

The authors declare that they have no competing interests.

## Authors' contributions

FMT conducted the study, analyzed the data and wrote the manuscript. AL and MF supervised the experiment, participated in the design of the study and revised the manuscript. CSL substantially contributed to statistical analysis of the data and editing of the paper. IL designed the study, performed preliminary analysis, helped to interpret the results and revised the manuscript. All authors have read and approved the final manuscript.
